# Reusing Plasma Filters in Resource-Poor Settings: Experience From a Tertiary Care Hospital

**DOI:** 10.7759/cureus.56516

**Published:** 2024-03-19

**Authors:** Priti Meena, Sandip panda, Paromita Das, Anish garg, Mohit Dayanandan

**Affiliations:** 1 Nephrology, All India Institute of Medical Sciences, Bhubaneswar, Bhubaneswar, IND

**Keywords:** cost reduction, guillain-barre syndrome (gbs), filter sterilization, dialysis, therapeutic plasma exchange (tpe)

## Abstract

Introduction: Therapeutic plasma exchange (TPE) is used to manage various life-threatening illnesses. It is widely performed by nephrologists, intensivists, pathologists, or experts in transfusion medicine worldwide. However, the costs of TPE sessions are exceedingly high, and they have a huge impact on patients' financial burden. Herein, we investigated the outcomes of the reuse of plasma filters in TPE on several occasions.

Methods: This is a retrospective analysis of patients receiving TPE from January 1, 2020, to April 30, 2023, in the Department of Nephrology. A formulation of 4.5% peracetic acid and 24% hydrogen peroxide acid with RO water dilution was used for reprocessing. Clinical outcomes, risks, and cost-benefit were evaluated and compared between the plasma filter reuse group (GP-1) and the no-reuse group (GP-2).

Results: A total of 70 patients were included in this study. 200 and 112 TPE sessions were performed in GP-1 and GP-2, respectively. The most common indication for TPE in both groups was neurological. The clinical efficacy of TPE was similar in both groups. There was no difference in the clotting of the plasma filter, any allergic reaction, infection, or bleeding in the group. However, there was a significant difference in levels of fibrinogen (p=0.03) pre and post-procedure in both groups. The incidence of hypotension was found to be higher in GP-1 (26%) compared to GP-2 (15.6%), p = 0.05. The cost of overall treatment was 38% less in GP-1.

Conclusion: The reuse of plasma filters is a safe and effective method for cost minimization in patients requiring TPE. This method can be effectively utilized in resource-poor settings without any increased risk of adverse effects.

## Introduction

Therapeutic plasma exchange (TPE) is used in the management of various life-threatening illnesses [1]. During TPE, plasma is separated from other constituents of blood (mostly cellular), and the removed plasma is then replaced by a replacement fluid, which is usually a colloid or sometimes a combination of crystalloid and colloid [2]. TPE can be used as a primary mode of management or as adjunctive therapy with other immunosuppressive agents [1]. It aids in the treatment of several illnesses occurring due to the presence of autoantibodies or immune activation leading to the formation of immune complexes and paraproteins. Especially in conditions like myasthenia gravis (MG), thrombotic thrombocytopenic purpura (TTP), treatment of renal allograft rejection, hemolytic uremic syndrome (HUS), and Guillain-Barre syndrome (GBS), TPE plays a cardinal role [3]. The membrane separator method for TPE is a commonly employed procedure extensively utilized by medical professionals specializing in nephrology, intensive care, and transfusion medicine worldwide. Nevertheless, the expenses associated with TPE sessions are exorbitant, resulting in a significant financial burden for patients. Most individuals are unable to afford the cost of a single session, which typically ranges from $1,200 to $1,500, and multiple sessions are often required. The expenditure associated with the administration of immunoglobulin (IVIG) further increases the overall cost of patient care [4]. This cost is too high for patients with economic hardship. The concept of reusing plasma filters during TPE has been examined previously but has never been implemented. The present study aimed to investigate the potential cost-saving benefits for patients through the analysis of the outcomes associated with the repeated use of plasma filters in TPE procedures.

## Materials and methods

Study design and setting

This is a retrospective analysis of patients receiving TPE from January 1, 2020, to April 30, 2023, in the Department of Nephrology. Our institute is one of the prime tertiary care hospitals in Eastern India. Patients older than 18 years undergoing TPE for various reasons were included in the study. Demographic data were collected: age, gender, indication for TPE, and comorbidities. Patients with hepatitis B and C, HIV, or symptoms or signs suggesting any infection were excluded. Laboratory parameters, including complete blood count, creatinine, electrolytes, serum albumin, prothrombin time (PT), International Normalized Ratio (INR), and activated partial thromboplastin time (APTT), were collected before and 30 minutes after the procedure. The number of reused plasmapheresis sessions and adverse effects were also noted. The policy of our institute mandates the acquisition of informed written consent prior to the commencement of TPE. The reuse of plasma filters was not employed in patients who did not give consent. An outcome analysis of clinical efficacy and adverse effects was performed to compare patients who underwent multiple uses of plasma filters (GP-1) versus those who underwent a single use of plasma filters (GP-2). A cost-benefit analysis was also conducted. The complications associated with vascular access have been described as pneumothorax, hemothorax, hematoma at the catheter site, thrombosis, and infection related to the catheter. The study has been approved by the ethical committee of the institute.

If a patient developed any febrile episode during the procedure, it was considered an infectious complication. Allergic episodes were considered if there was any episode of chill, rigor, asthma-like symptoms, skin rashes, back pain, or hypotension during the first 15 to 30 minutes of the procedure. Hypotension episodes were defined as a decrease in systolic blood pressure ≥20 mm Hg after initiating TPE. If the decrease in blood pressure was associated with clinical and nursing interventions, it was labeled as a symptomatic hypotensive episode.

Filter details

TPE was performed using the membrane separation method in all the patients. Fresenius Plasma Flux P2 was used in the study. It contains the polysulfone-based plasma sulfone membrane. Its effective surface area was 0.6 m^2^ with a max TMP of 100 mm Hg and a blood filling volume of 70 mL.

TPE procedure

The plasma filter was connected to the inlet and outlet of hemodialysis bloodlines. A double lumen central hemodialysis catheter or AV fistula was used as access. The procedure was performed with a pre-separator pump speed of 120-150 mL/min and pre-fractionator pump speed of 50-70 mL/min. Heparin was used for anticoagulation. At the onset of TPE, an initial bolus of 2000 units of heparin was given; thereafter, patients received a continuous infusion of 500 IU/hour. The plasma volume was estimated by the formula: Total body water × (1 - hematocrit) [5]. The replacement fluid was infused via the venous bloodline. Each session of TPE was usually completed in 2 to 2.5 hours.

Method of sterilization

The process of reusing the plasma filter was conducted manually by trained professionals in the Department of Nephrology of the hospital. After completing and initiating the TPE session with the patient, a careful inspection of the filter was conducted to check for any blood clots, contaminants, membrane ruptures, or irregularities by both doctors on duty in the dialysis department and the senior dialysis technician; if any were found, the filter was discarded. After mechanical cleaning with water treated by reverse osmosis under 15-20 psi pressure, each plasma filter was filled with a sterilizing agent [4.5% peracetic acid, 24% hydrogen peroxide] according to the manufacturer's instructions, the solution was diluted with water treated by reverse osmosis (RO) (100 mL of solution with 900 mL of RO water) [6,7]. The plasma filters were stored in individualized and clean boxes at room temperature until the next use (i.e., 48 to 72 hours later) [6]. The reuse of the resterilized filter was done in the same patient. The filters were labeled with the unique registration number and name of the patients after the procedure for reuse.

Statistical analysis

All statistical analyses were performed using IBM SPSS Statistics for Windows, Version 22 (Released 2013; IBM Corp., Armonk, New York). Means and medians with standard deviations (SD) were used for quantitative variables. Interquartile ranges (IQRs) were determined according to the distribution based on the Shapiro-Wilk test. Quantitative variables are also shown with frequencies and proportions. Comparisons of continuous variables were conducted using the Student t or Mann-Whitney tests. A p-value less than 0.05 was considered significant.

## Results

Patients

The study included 70 patients who fulfilled the inclusion criterion. Patients were categorized into two groups, GP-1 and GP-2, with 46 and 24 patients, respectively. The GP-1 group underwent 200 TPE sessions, while the GP-2 group underwent 112 TPE sessions. Among the cohort of 46 patients in GP-1, a single plasma filter was used on three separate occasions in six patients, while in the remaining cases, it was employed twice. N=43 (62%) of the total patients were male, and the median age was (IQR 32-64) years. Demographic and clinical details of the patients are presented in Table 1. In the majority of cases, a double-lumen hemodialysis catheter was the vascular access. The median number of TPE sessions per patient was 3 (IQR 1-6). The most frequent indication for plasmapheresis was neurological causes, with the majority of cases, n=13 (18.5%), being GBS followed by MG, neuromyelitis optica, and chronic inflammatory demyelinating polyneuropathy (CIDP).

**Table 1:** Baseline characteristics in the two groups GP-1 and GP-2 ANCA: antineutrophil cytoplasmic antibodies, GBM: glomerular basement membrane

Characteristics	GP-1 (N=46)	GP-2 (N=24)	p-value
Age in years ± SD	42 ± 15	39 ± 12	0.34
Gender, male n (%)	27 (58.7)	16 (66.6)	0.54
AV access			
Internal jugular catheter n (%)	34 (73.9)	19 (79.2)	0.15
AV fistula, n (%)	2 (4.4)	0	0.36
Femoral route catheter, n (%)	10 (21.7)	5 (20.8)	0.92
Average number of sessions per patient	4.7 ± 1.9 (range 1–6)	4.2 ± 2.2 (range 1–6)	0.42
Mean duration of each session (in minutes)	100 ± 30	96 ± 36	0.56
Mean volume of plasma extraction (in mL)	2200 ±400	2100 ±560	0.72
Mean hematocrit (%)	39.2 ± 3.4	38.2 ± 2.9	0.21
Mean serum albumin (g/dL)	4.2 ± 1.4	3.9 ± 1.8	0.29
Indications, n (%)			
Myasthenia gravis	6 (13.0)	3 (12.5)	0.34
Neuromyelitis optica	5 (10.8)	2 (8.3)	0.45
Guillain–Barré syndrome	8 (17.3)	5 (20.8)	0.76
Chronic inflammatory demyelinating polyneuropathy	3 (6.5)	3 (12.5)	0.26
Multiple sclerosis	2 (4.3)	3 (12.5)	0.51
Acute liver failures	4 (8.6)	2 (8.3)	0.98
Thrombotic thrombocytopenic purpura	3 (6.5)	2 (8.3)	0.71
Hemolytic uremic syndrome	1 (2.1)	–	
ANCA-associated vasculitis	3 (6.5)	–	
Anti-GBM disease	1 (2.1)	–	
Systemic lupus erythematosus	3 (6.5)	1 (8.3)	0.12
Allograft rejection	2 (4.3)	–	
Others	5 (10.8)	3 (12.5)	0.43

Efficacy

In both groups, the procedure was well tolerated. The response rates of diseases for which TPE was performed, in terms of improvement in clinical and biochemical parameters, were similar in both groups. Disease-specific scales such as the modified Rankin Scale (mRS) were used to assess clinical improvement in diseases like MG, GBS, and CIDP [8]. In cases of TTP and HUS, serum lactate dehydrogenase levels (LDH) <1.5 at the upper levels of the laboratory value and increment of platelet counts to >1.5 L per microliter was considered a complete response [7]. We did not find any difference in outcomes when the plasma filter was used in two or three times.

Coagulation parameters

On assessing the coagulation parameters measured pre- and post-TPE in both group procedures, the change in albumin (p=0.91), APTT (p=0.21), PT (p=0.08), serum IgG levels (p=0.07), and INR (p=0.45) was not statistically significant. However, the change in fibrinogen levels before and after TPE was 41% in GP-1 and 29% in GP-2, p=0.03, as shown in Table 2.

**Table 2:** Difference in the change in coagulation parameters pre- and post-TPE in both groups APTT: activated partial thromboplastin time, GP: group, INR: international normalized ratio, TPE: therapeutic plasma exchange

Characteristics	Before TPE	After TPE	p-value	Laboratory range
Albumin (GP-2)	3.43 ± 0.12	3.3 ± 0.24	0.91	3.5-5.2 g/dL
Albumin (GP-1)	3.432 ± 0.13	3.2 ± 0.21		
Serum IgG levels (GP-2)	8.60 ± 2.22	8.33 ± 1.9	0.07	7-16 g/L
Serum IgG levels (GP-1)	8.17 ± 1.7	6.98 ± 0.3		
INR (GP-2)	0.99 ± 0.05	1.03 ± 0.06	0.45	0.85–1.15
INR (GP-1)	0.96 ± 0.05	1.07± 0.03		
Prothrombin time (GP-2)	10.8 ± 0.60	11.2 ± 0.63	0.08	11-14 s
Prothrombin time (GP-1)	10.63 ± 0.62	11.63 ± 0.33		
APTT (GP-2)	21.7 ± 1.30	22.03 ± 1.17	0.21	26-38 s
APTT (GP-1)	22.9 ± 2.08	24.46 ± 1.67		
Fibrinogen (GP-2)	243.33 ± 31.05	172 ± 4.08	0.03	200-400 mg/dL
Fibrinogen (GP-1)	238 ± 85.09	140.3 ± 29.9		

Adverse events

The most common complication during the procedure was hypotension, as shown in Table 3. Incidences of hypotension were found to be higher in GP-1, n=12 (26%) compared to GP-2, n=4 (15.6%), p = 0.05. Most episodes, n=11 (91.6%) in GP-1 and n=3 (71%) in GP-2, of hypotension were asymptomatic, did not require ionotropic support or intensive care unit admission, and recovered after the procedure. The incidences of hypokalemia and hypocalcemia were also similar between the two groups. There was no difference in the rate of infections. No event of anaphylactoid reaction, vasovagal reaction, or death due to TPE was reported in any group. Our analysis did not find any increased evidence of adverse events in the reuse group.

**Table 3:** A comparison of adverse effects between groups 1 and 2. Vascular access complications are considered as pneumothorax, hemothorax, catheter site hematoma, thrombosis, and catheter-related infection. GP: group.

Characteristics	GP-1, n (%) (n=46)	GP-2, n (%) (n=24)	p-value
Vascular access complications	2 (4.3)	–	–
Fever	1 (2.1)	0	0.98
Chills	3 (6.5)	1 (4.1)	0.72
Abdomen pain	2 (4.3)	1 (4.1)	0.32
Vomiting	4 (8.7)	2 (8.3)	0.90
Headache	8	3 (12.5)	0.07
Hypotension	12 (26)	4 (15.6)	0.05
Bradycardia	4 (8.7)	2 (8.3)	0.22
Tachycardia	5 (10.8)	1(4.1)	0.54
Allergic reaction	0	0	–
Hemorrhage	0	0	–
Hypokalemia	4 (8.7)	2 (8.3)	0.51
Clotting of filter	7 (15.2)	3 (12.5)	0.21
Dyspnoea	1 (2.1)	0	–
Vasovagal reactions	0	0	–
Paraesthesia	6 (13)	3 (12.5)	0.43
Muscle spasm	9 (19.5)	3 (12.5)	0.27
Death	0	0	–

Cost-effectiveness

The expenses of five sessions of plasmapheresis in the GP-1 group were approximately half of those in the GP-2 group. One session of TPE costs 8,000-9,000 INR with a new plasma filter without IVIG and it becomes less than 1,000 INR with the reuse of a plasma filter. The cost of TPE without IVIG was 28,000-31,000 INR in GP-1 and 52,000-55,000 INR in GP-2. The hospital offers free provision of tubing, fresh frozen plasma, 5% albumin, and normal saline to patients. At our facility, there are no additional charges for the professional services rendered by physicians, nursing personnel, and technicians.

## Discussion

TPE is widely used in the management of immunologically mediated neurological diseases, as well as in non-neurological conditions such as hyperviscosity syndrome, TTP, HUS, vasculitis, and renal transplant rejections [1]. It is a common practice among nephrologists and is used to treat life-threatening illnesses. TPE can be done with either centrifugation or filtration. We studied the patients who received TPE through membrane filtration in the nephrology department.

The expense associated with a session of TPE in India exhibits significant variation among different medical facilities. This discrepancy is high when comparing private and government sectors, where the cost can range from 10,000 to 50,000 INR for a single TPE session. The expenses associated with this may be further increased with the use of albumin, fresh frozen plasma, and IVIG. The enormous cost of medical treatment with TPE poses a significant challenge for patients with constrained financial resources, impeding their ability to afford the necessary care and potentially resulting in deferred or delayed treatment. In certain instances, it may not be feasible for individuals to procure a new plasma filter for every session. The financial constraints faced by the patient in procuring the plasma filter, thereby impeding the commencement of TPE for a critical illness, promoted the consideration of the feasibility of reutilizing the filter for multiple sessions, provided it was in a suitable state, thereby obviating the need to halt the treatment. Previous studies have yielded some information concerning the reuse of plasma filters multiple times, but this practice is not usually carried out nowadays [7,9]. The current study evaluates the risks, benefits, and cost-effectiveness of the reuse of plasma filters compared to single-use TPE procedures. Consistent with other studies, the most common indication of TPE at our center was neurological disorders, followed by vasculitis [10,11]. The majority of patients belonged to the 30- to 40-year age group and were male. TPE-related complications were observed in 37.5% of patients overall in our study, without any statistically significant difference between both groups. There was no difference in clinical or serum markers related to the various diseases for which TPE was performed. No previous studies have explored the adverse effects associated with the reuse of plasma filters. Anticipated consequences include diminished efficacy, allergic reactions from sterilizing agents, infections, and clotting of the filters. In our study, we conducted an analysis of side effects. Notably, there were no reported deaths or allergic reactions across any study group. The analysis showed an increased occurrence of asymptomatic hypotension in the group subjected to filter reuse, and a decrease in plasma fibrinogen levels pre- and post-procedure in the reuse group.

Our study is one of the few that demonstrate the usefulness of reusing plasma filters on multiple occasions as a cost-effective approach for those who are economically disadvantaged and unable to purchase filters for each session. The limitations of our study include its retrospective nature and the fact that it was conducted at a single center. There may be selection bias when it comes to the criteria for TPE inclusion and the type of disease. We could not measure the concentration of molecules like albumin or immunoglobulins in the effluent to assess the filter's performance with subsequent uses [12]. Although it was not possible to evaluate the clinical efficacy of TPE for each disorder, it has been utilized whenever feasible, such as in neurological diseases where a scale is applicable. In cases involving hematological disorders, laboratory parameters, including LDH and platelets, have been utilized. Nonetheless, given the multitude of variables that influence disease recovery, it is not possible to unequivocally establish the effectiveness of TPE in improving the disease. Deciphering the likely causes behind the elevated occurrences of hypotension and notable variations in fibrinogen levels between fresh and reused filters poses a challenge. The observed outcomes were unexpected, prompting a thorough exploration of potential explanations. Among the conceivable reasons, residual chemical infusion (Rebound Release) emerges as the most plausible hypothesis. The manifestation of rebound release implies the disinfectant's binding to a membrane or another component of the filter, with a delayed release during the procedure, potentially contributing to hypotensive episodes. 

## Conclusions

In conclusion, this retrospective analysis conducted at our tertiary care hospital in eastern India highlights the feasibility and safety of reusing plasma filters in TPE procedures. The study, spanning from January 2020 to April 2023, included 70 patients with varying indications for TPE. The results demonstrate comparable clinical efficacy between the group using reused plasma filters (GP-1) and the single-use group (GP-2). A dilution of 4.5% peracetic acid and 24% hydrogen peroxide acid with RO water was used for reprocessing. The incidence of hypotension was higher in GP-1, but these episodes were mostly asymptomatic and self-resolving. Importantly, the cost-benefit analysis revealed a substantial reduction in the overall treatment cost in GP-1, suggesting the economic viability of plasma filter reuse without compromising patient outcomes. While the study offers promising insights, further research, including randomized control trials with larger cohorts, is imperative to validate these findings and establish widespread applicability.

## References

[1] Fernández-Zarzoso M, Gómez-Seguí I, de la Rubia J (2019). Therapeutic plasma exchange: review of current indications. Transfus Apher Sci.

[2] Cervantes CE, Bloch EM, Sperati CJ (2023). Therapeutic Plasma Exchange: Core Curriculum 2023. Am J Kidney Dis.

[3] Padmanabhan A, Connelly-Smith L, Aqui N (2019). Guidelines on the use of therapeutic apheresis in clinical practice - evidence-based approach from the writing committee of the American Society for Apheresis: the eighth special issue. J Clin Apher.

[4] Maheshwari A, Sharma RR, Prinja S, Hans R, Modi M, Sharma N, Marwaha N (2018). Cost-minimization analysis in the Indian subcontinent for treating Guillain Barre syndrome patients with therapeutic plasma exchange as compared to intravenous immunoglobulin. J Clin Apher.

[5] Kaplan AA (1990). A simple and accurate method for prescribing plasma exchange. ASAIO Trans.

[6] Schoenfeld PY (1997). The technology of dialyzer reuse. Semin Nephrol.

[7] Houssin A, Champion G, Hurez D (1982). Performance of reused plasma separating filters (Article in French). Pathol Biol (Paris).

[8] Farrugia ME, Carmichael C, Cupka BJ, Warder J, Brennan KM, Burns TM (2014). The modified Rankin scale to assess disability in myasthenia gravis: comparing with other tools. Muscle Nerve.

[9] Smetisko A, Slavas-Boras S, Thune S, Grgas J, Puretić Z, Bratelj Z, Gabrić V (1985). Possibilities of plasma filter's reuse. Przegl Lek.

[10] Cuker A, Cataland SR, Coppo P (2021). Redefining outcomes in immune TTP: an international working group consensus report. Blood.

[11] Shemin D, Briggs D, Greenan M (2007). Complications of therapeutic plasma exchange: a prospective study of 1,727 procedures. J Clin Apher.

[12] Bobati SS, Naik KR (2017). Therapeutic plasma exchange - an emerging treatment modality in patients with neurologic and non-neurologic diseases. J Clin Diagn Res.

